# Flock Management Risk Factors Associated with Q Fever Infection in Sheep in Saudi Arabia

**DOI:** 10.3390/ani11071948

**Published:** 2021-06-30

**Authors:** Ibrahim Elsohaby, Ahmed Elmoslemany, Mohamed El-Sharnouby, Mohamed Alkafafy, Mohammed Alorabi, Wael M. El-Deeb, Theeb Al-Marri, Ibrahim Qasim, Fanan A. Alaql, Mahmoud Fayez

**Affiliations:** 1 Department of Animal Medicine, Faculty of Veterinary Medicine, Zagazig University, Zagazig City 44511, Egypt; 2 Department of Health Management, Atlantic Veterinary College, University of Prince Edward Island, Charlottetown, PE C1A 4P3, Canada; 3 Hygiene and Preventive Medicine Department, Faculty of Veterinary Medicine, Kafrelsheikh University, Kafr El-Sheikh 33516, Egypt; aelmoslemany@gmail.com; 4 Department of Biotechnology, College of Science, Taif University, P.O. Box 11099, Taif 21944, Saudi Arabia; m.sharnouby@Tu.edu.sa (M.E.-S.); m.kafafy@tu.edu.sa (M.A.); maorabi@tu.edu.sa (M.A.); 5 Department of Clinical Sciences, College of Veterinary Medicine, King Faisal University, Al-Ahsa 31982, Saudi Arabia; weldeeb@kfu.edu.sa; 6 Department of Veterinary Medicine, Infectious Diseases and Fish Diseases, Faculty of Veterinary Medicine, Mansoura University, Mansoura 35516, Egypt; 7 Al Ahsa Veterinary Diagnostic Lab, Ministry of Environment, Water and Agriculture, Al-Ahsa 31982, Saudi Arabia; theep8@hotmail.com (T.A.-M.); mahmoudfayez30@hotmail.com (M.F.); 8 Ministry of Environment, Department of Animal Resources, Water and Agriculture, Riyadh 12629, Saudi Arabia; i.qasim@mewa.gov.sa; 9 Department of Microbiology, College of Science, King Saud University, Riyadh 11451, Saudi Arabia; fanan.abdulaziz@gmail.com; 10 Department of Bacteriology, Veterinary Serum and Vaccine Research Institute, Ministry of Agriculture, Cairo 11381, Egypt

**Keywords:** Q fever, risk factors, sheep, ticks, ELISA, abortion, multivariable analysis

## Abstract

**Simple Summary:**

Q fever is a zoonotic disease with significant public health implications. Sheep are one of the main reservoirs for this disease, whereas abortion is the primary clinical outcome. Q fever is endemic in many countries, including Saudi Arabia. Flock management practices play a significant role in the spread of Q fever infection among flocks. However, information on flock management factors associated with Q fever seropositivity in Saudi Arabia is very scarce. The results obtained from 50 flocks identified three protective factors (lambing pen, change bedding after removing aborted materials, and isolation of aborted ewes) and two risk factors (infestation with ticks and history of Q fever) were associated with Q fever seropositivity.

**Abstract:**

Q fever is a zoonotic disease caused by *Coxiella burnetii* (*C. burnetii*), an intracellular, Gram-negative bacterium that infects humans and domestic ruminants. Information on flock management factors associated with Q fever seropositivity in Saudi Arabia is very scarce. Therefore, the objective of this study was to identify the animal and flock management factors associated with Q fever seropositivity. For the assessment of risk factors, a case-control study was carried out. Cases (*n =* 25) were flocks that had recent abortions within the previous two weeks and were PCR positive for *C. burnetii*. Control flocks (*n =* 25) had no history of recent abortion and were PCR negative for *C. burnetii*. A questionnaire was developed to collect information about the flock management risk factors possibly associated with Q fever exposure in sheep. A total of 2437 sheep serum samples, collected from infected (*n* = 1610, 10–150 samples/flock) and non-infected (*n* = 827, 10–65 samples/flock) flocks, were tested for *C. burnetii* antibodies using a commercial ELISA kit between May 2018 and April 2019. In addition, 521 samples, including 50 aborted materials, 173 vaginal swabs, 134 faecal, and 164 milk samples, were collected for PCR testing. Infected flocks were 100% seropositive (within-flock seroprevalence ranging between 13.8% and 60%) and 100% PCR positive (with animal shedders of *C. burnetii* through aborted materials and/or vaginal fluids, feces, and milk). However, in non-infected control flocks, 28% were seropositive (within-flock seroprevalence ranging between 6.7% and 20%) and none had *C. burnetii* shedders. Epidemiological data were analyzed using mixed-effect logistic regression with a random effect for the flock. The results identified three protective factors: flocks with a lambing pen (odds ratio (OR): 0.46; 95% CI: 0.28–0.76), change bedding after removing aborted materials (OR: 0.42; 95% CI: 0.23–0.76), and flocks that isolated aborted ewes (OR: 0.41; 95% CI: 0.25–0.67), as well as two risk factors: flocks infested with ticks (OR: 2.78; 95% CI: 1.65–4.70) and flocks with a history of Q fever (OR: 3.03; 95% CI: 1.42–6.50). These results could be used to improve sheep flock biosecurity measures to prevent the introduction and reduce exposure of sheep and humans to Q fever infection.

## 1. Introduction

Q fever is an important zoonotic disease with great public health consideration throughout the world. The causative agent is *Coxiella burnetii* (*C. burnetii*), which is an obligated intracellular Gram-negative bacterium [1]. *Coxiella burnetii* has a spore-like structure and can survive harsh environmental conditions [2]; thus, the Centers for Disease Control and Prevention (CDC) classifies *C. burnetii* as a Category B pathogen with potential use for biological weapons [3].

*Coxiella burnetii* can infect humans and a wide range of mammalian and non-mammalian animals [4]. However, domestic ruminants (sheep, goat and cattle) are considered the primary reservoir of *C. burnetii* [5,6]. Other animals including dogs, cats, rabbits, and birds are susceptible and could transfer Q fever to humans [7]. Moreover, ticks and rodents are also a natural reservoir of *C. burnetii* [8]. Infected animals shed *C. burnetii* in feces, urine, milk, colostrum, placenta, vaginal excretion, and uterine fluids [9,10]. However, the main route of *C. burnetii* infection in both humans and animals is the inhalation of infectious aerosols or dust [11,12]. Furthermore, ticks play a significant role in maintaining *C. burnetii* infection among animals, especially within the sylvatic cycle that involves wild animals [13].

Q fever infection in ruminants is usually subclinical; however, reproductive disorders are the serious clinical outcome of such infection [14]. *Coxiella burnetii* infection in sheep is mostly asymptomatic, but it can cause abortion in late gestation, stillbirth, premature lambing, and delivery of weak lamb [9,10]. In humans, Q fever is considered an occupational disease and most of the outbreaks were reported among veterinarians, livestock breeders, butchers, abattoir, and farm workers [15]. The disease in humans is characterized by fever, flu-like symptoms, and headache, and some cases may suffer from pneumonia, hepatitis, and endocarditis [7,16,17].

Different flock-level managemental factors, including flock density and contact with farm visitors, were associated with higher Q fever seropositivity [18]. Previous studies have reported that the real drivers of Q fever infection in sheep, goat, and cattle flocks/herds were intrinsic farm factors such as production system and management practices [19,20].

In Saudi Arabia, Q fever is an endemic disease, and several seroprevalence surveys have been conducted in various ruminants, including camels [21,22], cattle, sheep, and goats [23,24]. Although determining farm-level risk factors for Q fever infection is important for controlling infection in both animals and humans, none of the former studies investigated the association between farm management factors and *C. burnetii* infection in sheep in Saudi Arabia. Therefore, the objective of the present study was to identify the risk factors associated with exposure to Q fever among sheep flocks in Eastern Province, Saudi Arabia.

## 2. Materials and Methods

### 2.1. Study Area and Flock Management

**Figure 1:**
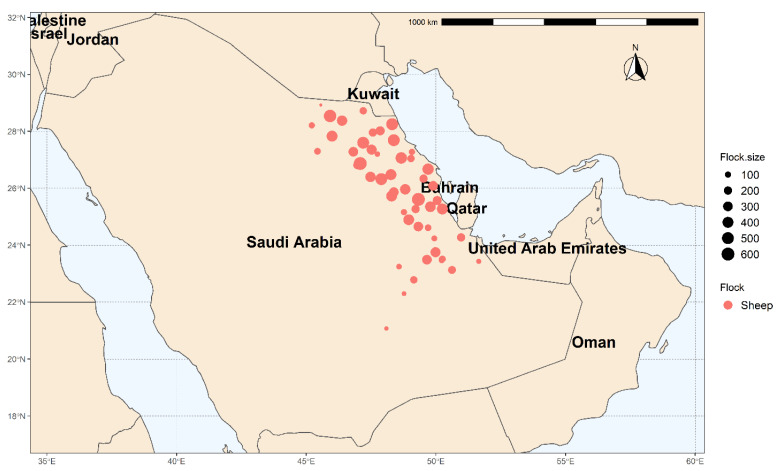
The location and size of tested flocks in Eastern Province, Saudi Arabia.

The present study was carried out in the Eastern Province of Saudi Arabia, which is located at 22°30′ N, 51°00′ E, at 390 km from the capital Riyadh (Figure 1). The Eastern Province is Saudi Arabia’s third most populated province, having a variety of climates ranging from semi-desert to desert. In addition, the Eastern Province is bordered by five countries (Iraq, Kuwait, Oman, Qatar, and UAE), which may increase the possibility of pathogen introduction into the region. According to the General Statistical Authority for 2015, 13% (over 4 million) of the Kingdom’s overall sheep population are in the Eastern Province.

Fifty flocks located in the Eastern Province of Saudi Arabia were involved in the present study from May 2018 to April 2019. The flock size varied from 40 to 600 sheep (media *n* = 270), and all flocks were for meat production. The most common breed is Najdi (*n* = 18), followed by Awassi (*n* = 16), Sawakni (*n* = 9), and Harri (*n* = 7). The production system is mostly seminomadic, with sheep grazing away from their raising farms when pastures are available (from October to April). However, from May to September, sheep are housed and fed ad libitum, barley, wheat bran, and straw.

### 2.2. Study Design and Sampling Process

A case-control study was carried out to evaluate the association between flock management risk factors and *C. burnetii* infection. Cases (*n* = 25) were flocks that had recent abortions within the previous two weeks and with at least one animal PCR positive for *C. burnetii*. Control flocks (*n* = 25) had no history of recent abortion and were PCR negative for *C. burnetii*.

Flock selection: Cases were selected from aborted cases submitted to the Veterinary Diagnostic Laboratory after excluding other causes of abortion. Aborted flocks were first screened for brucellosis using the Rose Bengal test; negative flocks were then tested for *C. burnetii* and *Chlamydia abortus* using ELISA. Case flocks were then visited for further sampling and risk factor assessment. Finally, control flocks were selected from flocks submitting samples for diagnostic reasons that were not related to abortion.

Selection of animals within flocks: The minimum number of animals required for detection of infection within flocks were determined using the following formula [25]:

*n =* {1 − (1 − P1) ^1/d^} {N − d/2} + 1 (1)

where *n* = required sample size; P1 = probability of finding at least one case in the sample (0.95); d = minimum number of affected animals expected in the flock (assuming 15% seroprevalence); and N = flock size.

PCR samples (vaginal swabs, fecal, and milk) were collected from adult sheep for both case and control flocks. Additionally, aborted materials were collected from case flocks when possible. In total, 521 samples (aborted materials (*n* = 50), vaginal swabs (*n* = 173), faecal (*n* = 134), and milk (*n* = 164) samples) were collected from all flocks for PCR testing and at least one sample was collected from each flock. Additionally, for ELISA testing, blood samples were collected from 2437 sheep from the 50 study flocks. All samples were labelled with flock and sheep identification numbers as well as sampling times and then transported to the laboratory for serological examination.

### 2.3. Epidemiological Data Collection

A standardized questionnaire was created to gather data on flock management risk factors that may be linked to Q fever exposure in sheep. The questionnaire, which was written in Arabic and consisted of 15 closed and open-ended questions, is accessible upon request from the corresponding author. On the day of sampling, face-to-face interviews with the flock owner were used to complete the questionnaire. Questions covered the flock characteristics and management practices, including flock size, purchase of a breeding replacement, quarantine of purchased animals, contact with other sheep flocks or other animals, presence of ticks on the animals or environment, animal exchange during breeding, manure spreading, presence of lambing pen, changing and disinfection of bedding after abortion, isolation of aborted ewes, and recent history of abortion and Q fever.

### 2.4. Serological Test

Commercial indirect ELISA kits (IDEXX Q Fever Ab Test, IDEXX Laboratories, Broomfield, CO, USA) were used to test serum samples for antibodies against *C. burnetii* infection according to the manufacturer’s instructions. Briefly, serum and control tests were diluted at 1:400, and tested in duplicates. The optical densities (ODs) were determined at 450 nm using a microplate ELISA reader. The average OD of the duplicate samples and controls was calculated and then used to estimate the OD% by the following equation:

OD (%) = (OD _sample_ − OD _negative control_)/(OD _positive control_ − OD _negative control_) × 100. (2)

Samples were classified as seropositive if the OD% > 40% and seronegative if the OD% < 30%. However, samples with OD% between ≥30% and ≤40% were classified as doubtful.

### 2.5. Molecular Identification of C. burnetii

Aborted materials, vaginal, faecal, and milk samples were decontaminated with ethanol 70%, and then inactivated at 90 °C for 20 min to ensure the safety of laboratory personnel prior to DNA extraction. The genomic DNA was extracted using the QIAamp DNA Mini-Kit (Qiagen SA, Courtaboeuf, France) following the manufacturer’s instructions. A specific primer (forward 5′-GTCTTAAGGTGGGCTGCGTG-3′, reverse 5′-CCCCGAATCTCATTGATCAGC-3′) was used to amplify a 290 bp fragment from the repetitive element IS1111a (Transposase) of the *C. burnetii* genome. The amplified fragment was detected using a probe labelled with LightCycler^®^ Red 640 (5′-GTTACTTTTGACATACGGTTTGACGTGCT-3′) (TIB Molbiol, Berlin, Germany). The PCR reagents used to prepare the reaction mix were from Roche Diagnostics (Penzberg, Germany). The PCR amplification was performed in LightCycler^®^ 2 as described by Stemmler and Meyer [26]. Positive and negative controls were included in each PCR reaction. Samples with Ct value between 17 and 35 were considered positive.

### 2.6. Statistical Analysis

For descriptive and statistical data analysis, Stata Statistical Software v.16 (Stata Corp, College Station, TX, USA) was used and the results were considered significant at *p*-value < 0.05. In order to investigate the possible risk factors for Q fever seropositivity, univariable and multivariable logistic regression models with random effects were applied. Firstly, univariable analysis was used to screen all risk factors for unconditional association with Q fever seropositivity at liberal *p*-value < 0.20. Significant factors were checked for collinearity using Spearman correlation coefficients and considered collinear if the coefficient was >0.8 [27].

Multivariable logistic regression was performed using variables retained from the univariable analysis, and non-significant variables were excluded sequentially using backward elimination at *p*-value < 0.05. The significance of two-way interactions among biologically relevant variables in the final main effect model was assessed. Eliminated variables were checked for confounding and were kept in the model if their inclusion shifted the coefficients of one or more significant variables by more than 20% [28].

## 3. Results

### 3.1. Description of ELISA and PCR Results

Infected flocks were 100% seropositive (within-flock seroprevalence ranging between 13.8% and 60%) and 100% PCR positive (with animal shedders of *C. burnetii* through aborted materials and/or vaginal fluids, feces, and milk). However, in non-infected control flocks, 28% were seropositive (within-flock seroprevalence ranging between 6.7% and 20%) and none had *C. burnetii* shedders. In infected flocks, the percentage of PCR positive samples was greater in samples from aborted materials (88.9%) and vaginal swabs (56.6%) compared with faecal (25.6%) and milk samples (25.3%). The number of tested samples and proportion of Q fever positive samples in each infected and non-infected control flock are summarized in Supplementary Table S1. Furthermore, the management practices of Q fever infected and non-infected control flocks are described in Supplementary Table S2.

### 3.2. Risk Factor Analysis

The flock management factors associated with sheep seropositivity in a univariable analysis at *p* < 0.20 are presented in Table 1. The results indicated a significant positive association (risk factor) with larger flock size, purchase of a breeding replacement, animal exchange during breeding, contact with other sheep flocks or other animals, presence of ticks on the animals or environment, manure spreading, and recent history of abortion and Q fever. However, factors such as quarantine of purchased animals, having lambing pen, change of bedding after removal of aborted materials, bedding disinfection after abortion, and isolation of aborted ewes were negatively associated (protective factors) with Q fever seropositivity.

Table 2 shows flock management factors associated with sheep seropositivity in a multivariable analysis at *p* < 0.05. Only five factors were considered to be significantly associated with Q fever seropositivity under the final multivariable logistic regression model. The results showed that flocks with lambing pen, flocks changing bedding after abortion, and flocks that isolated aborted ewes from healthy ewes were 0.46, 0.42, and 0.41 times less likely to be seropositive for Q fever compared with flocks without lambing pens, flocks that do not change bedding material after abortion, and flocks that do not isolate aborted animals from the rest of the flock, respectively. On the other hand, the presence of ticks in the environment was associated with a higher risk of seropositive status for Q fever (~3 times higher). Finally, flocks with a history of Q fever were three times more likely to be seropositive for Q fever compared with other flocks.

## 4. Discussion

In this study, we investigated the exposure and infection of sheep flocks with a history of abortion to Q fever by testing for the presence of *C. burnetii* with an indirect ELISA test and PCR. In this study, the proportion of seropositive sheep within flocks ranged from 6.7% to 60%, indicating the widespread nature of *C. burnetii* within sheep flocks in the Eastern Province, Saudi Arabia. Sheep flocks in Saudi Arabia are seminomadic and move for long distances every day during the grazing season, allowing *C. burnetii* to spread horizontally to many flocks. The highest proportion of seropositive sheep within flock reported in this study was 60%, recorded in the large-sized flocks and confirmed by the univariable logistic analysis. This finding is in line with recent studies from the United Kingdom [29] and Canada [30], indicating that larger flocks are linked to higher Q fever seropositivity than smaller flocks. The possible explanation is that the overcrowding of sheep in larger flocks may impact animal welfare and hygienic measures, which increase the risk of disease transmission. In addition, previous work in Q fever in goats and cattle indicated that larger herd sizes would result in a larger degree of environmental contamination with *C. burnetii* [31,32]. The association between a higher seropositivity rate and a greater flock size may also be explained by the increased number of veterinarians, feed providers, and farm staff visiting the farms [20,33,34].

Several studies have pointed out that the proportion of Q fever infection in small ruminants differs with the management system operated on the flock [20,30]. As a result, we can conclude that flock management practices in Saudi Arabia play a significant role in the high proportion of Q fever seropositivity recorded in the present study. The univariable risk factor analysis of the flock management showed many factors to be significantly (*p* < 0.20) associated with Q fever seropositivity. The contact with other animals and sheep flocks and purchased breeding replacements were identified as risk factors for *C. burnetii* infection and support the spread of infection from one flock to another, indicating the lack of biosecurity measures and explaining the high flock seropositivity reported in this study. Similar findings have previously been found in sheep flocks in India [35], Algeria [36], and Canada [30]. Furthermore, it was revealed that the proportion of sheep exposed to Q fever in the present study significantly decreases in flocks where quarantine of newly purchased animals, change and disinfection of bedding, and removal of aborted materials were applied. This is in agreement with the study of Meadows et al. [30], who reported that changing and disinfection of lambing bedding may be an effective tool for reducing *C. burnetii* infection. In addition, it has been reported that the lack of quarantine of newly purchased animals increases the risk of *C. burnetii* infection [37]. *Coxiella burnetii* can persist in harsh environmental conditions and survive for a long time [20,30]. Therefore, flocks spreading manure had a high proportion of Q fever infected sheep, which may be attributed to the contamination of the environment with *C. burnetii*.

The risk of sheep flocks acquiring Q fever seems to be complex. The multivariable analysis in this study indicated that the Q fever seropositivity was associated with five different variables. Flocks having lambing pens, change bedding after abortion, and isolating aborted ewes were identified as factors that protect against Q fever infection. As lambing area, bedding contaminated with aborted materials, and aborted ewes are likely a heavy source of *C. burnetii* [38], the isolation of lambing pen, aborted ewes, and removal of contaminated bedding from the direct contact with other animals in the flock decrease the risk of *C. burnetii* infection [30]. A high proportion of Q fever infected sheep was observed in flocks with a history of Q fever infection, in contrast to flocks with low rates of Q fever infection. These findings were consistent with previous studies linking abortions to Q fever [39,40,41].

The role of ticks in the transmission and spread of *C. burnetii* infection has been reported [42,43,44]. An infected tick can directly transmit *C. burnetii* infection to sheep. Furthermore, previous studies have reported that tick feces containing high amounts of viable *C. burnetii* could be indirectly transmitted to sheep and humans by inhalation [45]. In the present study, the risk of Q fever seropositivity in flocks infested with ticks is higher compared with flocks not infested with ticks, which is consistent with previous reports [43,44,46]. In contrast, other studies reported a low risk of *C. burnetii* infection in flocks infested with ticks in some endemic areas in Europe [47,48].

## 5. Conclusions

A high proportion of Q fever infection among sheep flocks with a history of abortion in the Eastern Province, Saudi Arabia, was observed in the present study. As a result, certain management practices, especially those identified as protective risk factors in this work, such as having a lambing pen, changing bedding contaminated with aborted materials, and isolation of aborted ewes, must be implemented. Furthermore, control of ticks on animals and farm environments could reduce the spread of *C. burnetii* infection between flocks and possible transmission to humans.

This finding can be used to reinforce the importance of hygiene and biosecurity measures in mitigating Q fever infection among sheep flocks and humans. Thus, farmers, veterinarians, and epidemiologists should work together to implement effective management interventions to control Q fever.

## Figures and Tables

**Table 1:** Univariable analysis of flock management risk factors association (*p* < 0.20) with Q fever seropositivity among sheep sampled from 50 flocks in Eastern Province, Saudi Arabia.

Factors	Frequency of Examined Sheep (%)	Proportion of Seropositive Sheep (%)	OR (95% CI) ^1^	*p-*Value
Flock size				
Small < 290	24.74	11.28	1.00 (ref.)	0.177
Medium (290–500)	46.94	15.56	1.62	0.353
Large > 500	28.31	19.13	3.83	0.065
Purchase of breeding replacement				
No	27.62	12.78	1.00 (ref.)	
Yes	72.38	16.55	3.30	0.024
Quarantine of purchased animals				
No	66.03	18.38	1.00 (ref.)	
Yes	33.97	13.92	0.53	0.141
Animal exchange during breeding				
No	34.76	8.38	1.00 (ref.)	
Yes	65.24	19.31	5.67	0.000
Contact with other sheep flocks				
No	25.81	8.90	1.00 (ref.)	
Yes	74.19	17.81	3.03	0.024
Contact with other animals				
No	12.11	5.08	1.00 (ref.)	
Yes	87.89	16.95	7.40	0.006
Lambing pen				
No	52.03	19.16	1.00 (ref.)	
Yes	47.97	11.55	0.28	0.005
Recent history of abortion				
No	18.59	4.19	1.00 (ref.)	
Yes	81.41	18.09	7.08	0.0001
Change bedding after removing aborted materials				
No	57.20	18.72	1.00 (ref.)	
Yes	42.80	11.22	0.26	0.003
Disinfect bedding after abortion				
No	77.51	18.26	1.00 (ref.)	
Yes	22.49	6.02	0.20	0.001
Isolate aborted ewes				
No	56.65	22.15	1.00 (ref.)	
Yes	43.35	12.79	0.32	0.001
Ticks on animals				
No	45.88	12.52	1.00 (ref.)	
Yes	54.12	18.04	2.85	0.029
Ticks in environment				
No	67.21	13.49	1.00 (ref.)	
Yes	32.79	19.65	3.50	0.014
Manure spreading				
No	31.56	9.23	1.00 (ref.)	
Yes	68.44	18.41	5.13	0.001
History of Q fever				
No	87.48	14.40	1.00 (ref.)	
Yes	12.52	23.28	5.10	0.038

^1^ OR: odds ratio; CI: confidence interval.

**Table 2:** Multivariable logistic regression analysis of flock management risk factors association (*p* < 0.05) with Q fever seropositivity among sheep sampled from 50 flocks in Eastern Province, Saudi Arabia.

Factors	OR (95% CI) ^1^	*p-*Value
Lambing pen		
No	1.00 (ref.)	
Yes	0.46 (0.28–0.76)	0.002
Change bedding after removing aborted materials		
No	1.00 (ref.)	
Yes	0.42 (0.23–0.76)	0.004
Isolate aborted ewes		
No	1.00 (ref.)	
Yes	0.41 (0.25–0.67)	0.000
Ticks in environment		
No	1.00 (ref.)	
Yes	2.78 (1.65–4.70)	0.000
History of Q fever		
No	1.00 (ref.)	
Yes	3.04 (1.42–6.50)	0.004
**Random Effects Parameters**	**Estimate (SE)**	***p-*Value**
Flock	0.24 (0.14)	0.001

^1^ OR: odds ratio; CI: confidence interval; SE: standard error.

## Data Availability

The data presented in this study are available on request from the corresponding author.

## Supplementary Materials

The following are available online at https://www.mdpi.com/article/10.3390/ani11071948/s1, Table S1. Total number of tested and proportion of Q fever positive sheep by ELISA and PCR in infected and non-infected control flocks. Table S2. Management practices of Q fever infected and non-infected control flocks in Eastern Province, Saudi Arabia.
