# Supplementary material for: Flock Management Risk Factors Associated with Q Fever Infection in Sheep in Saudi Arabia
Source: Animals (Basel). 2021 Jun 30;11(7):1948. doi: 10.3390/ani11071948 (PMC8300262; doi:10.3390/ani11071948)
Supplement: Supplementary file 1 [file animals-11-01948-s001.zip › animals-1279086-SI.pdf]

Table S1. Total number of tested and proportion of Q fever positive sheep by ELISA and PCR in infected and non-infected control flocks.

Flock ID	Tested flocks		No. of tested sheep (% of positive)				
	ELISA	PCR	ELISA (Serum)	PCR (Aborted materials)	PCR (Vaginal swabs)	PCR (Faecal)	PCR (Milk)
**I. Flocks with cases (n = 25)**							
2	+	+	20 (15.0)	0 (0.0)	2 (100.0)	0 (0.0)	3 (0.0)
3	+	+	20 (30.0)	1 (100.0)	5 (60.0)	0 (0.0)	2 (0.0)
6	+	+	40 (20.0)	1 (100.0)	2 (50.0)	3 (66.7)	4 (25.0)
8	+	+	35 (28.6)	2 (100.0)	5 (60.0)	2 (50.0)	3 (66.7)
11	+	+	80 (27.5)	3 (100.0)	6 (66.7)	5 (40.0)	4 (25.0)
13	+	+	20 (45.0)	2 (100.0)	6 (33.3)	2 (0.0)	2 (0.0)
15	+	+	60 (23.3)	2 (100.0)	5 (60.0)	4 (25.0)	3 (33.3)
18	+	+	75 (30.7)	1 (100.0)	3 (100.0)	3 (33.3)	3 (33.3)
19	+	+	10 (60.0)	1 (100.0)	3 (66.7)	1 (100.0)	0 (0.0)
20	+	+	100 (20.0)	1 (100.0)	4 (50.0)	3 (0.0)	4 (50.0)
22	+	+	85 (22.4)	2 (100.0)	5 (60.0)	3 (33.3)	3 (33.3)
27	+	+	55 (14.5)	1 (100.0)	4 (25.0)	2 (0.0)	4 (0.0)
28	+	+	130 (23.1)	1 (100.0)	4 (25.0)	3 (0.0)	6 (16.7)
30	+	+	15 (40.0)	1 (100.0)	4 (25.0)	3 (0.0)	3 (0.0)
36	+	+	25 (28.0)	1 (100.0)	3 (100.0)	3 (0.0)	3 (0.0)
37	+	+	110 (23.6)	3 (66.7)	5 (40.0)	4 (50.0)	4 (50.0)
39	+	+	20 (25.0)	1 (100.0)	5 (40.0)	4 (0.0)	5 (0.0)
41	+	+	150 (16.0)	2 (100.0)	5 (100.0)	5 (40.0)	6 (33.3)
43	+	+	40 (20.0)	0 (0.0)	3 (33.3)	4 (0.0)	4 (25.0)
44	+	+	80 (13.8)	1 (100.0)	4 (50.0)	5 (40.0)	5 (0.0)
45	+	+	140 (19.3)	2 (100.0)	4 (50.0)	5 (20.0)	5 (40.0)
46	+	+	100 (14.0)	1 (100.0)	5 (20.0)	4 (25.0)	5 (0.0)
48	+	+	50 (30.0)	3 (0.0)	7 (57.1)	4 (50.0)	4 (100.0)
49	+	+	60 (15.0)	1 (100.0)	3 (100.0)	6 (16.7)	6 (16.7)
50	+	+	90 (23.3)	2 (100.0)	4 (100.0)	4 (25.0)	4 (25.0)
**Total**	**25 (100.0)**	**25 (100.0)**	**1610 (21.8)**	**36 (88.9)**	**106 (56.6)**	**82 (25.6)**	**95 (25.3)**
**II. Control flocks (n = 25)**							
1	−	−	10 (0.0)	0 (0.0)	1 (0.0)	0 (0.0)	1 (0.0)
4	−	−	20 (0.0)	0 (0.0)	2 (0.0)	0 (0.0)	3 (0.0)
5	+	−	25 (6.7)	0 (0.0)	3 (0.0)	0 (0.0)	3 (0.0)
7	−	−	40 (0.0)	2 (0.0)*	3 (0.0)	1 (0.0)	3 (0.0)

9	−	−	30 (0.0)	0 (0.0)	3 (0.0)	2 (0.0)	2 (0.0)
10	−	−	40 (0.0)	2 (0.0)*	5 (0.0)	2 (0.0)	5 (0.0)
12	+	−	24 (16.7)	0 (0.0)	3 (0.0)	2 (0.0)	3 (0.0)
14	−	−	19 (0.0)	2 (0.0)*	3 (0.0)	2 (0.0)	2 (0.0)
16	−	−	45 (0.0)	0 (0.0)	2 (0.0)	2 (0.0)	2 (0.0)
17	−	−	15 (0.0)	0 (0.0)	3 (0.0)	3 (0.0)	2 (0.0)
21	−	−	50 (0.0)	1 (0.0)*	3 (0.0)	2 (0.0)	3 (0.0)
23	+	−	30 (20.0)	0 (0.0)	3 (0.0)	3 (0.0)	4 (0.0)
24	−	−	30 (0.0)	0 (0.0)	2 (0.0)	2 (0.0)	2 (0.0)
25	−	−	25 (0.0)	0 (0.0)	3 (0.0)	2 (0.0)	3 (0.0)
26	−	−	45 (0.0)	0 (0.0)	3 (0.0)	2 (0.0)	3 (0.0)
29	−	−	25 (0.0)	0 (0.0)	3 (0.0)	3 (0.0)	3 (0.0)
31	−	−	10 (0.0)	2 (0.0)*	2 (0.0)	2 (0.0)	3 (0.0)
32	−	−	50 (0.0)	0 (0.0)	3 (0.0)	3 (0.0)	3 (0.0)
33	+	−	60 (6.7)	0 (0.0)	2 (0.0)	2 (0.0)	2 (0.0)
34	−	−	20 (0.0)	0 (0.0)	2 (0.0)	2 (0.0)	2 (0.0)
35	+	−	34 (11.8)	0 (0.0)	3 (0.0)	3 (0.0)	3 (0.0)
38	−	−	50 (0.0)	2 (0.0)*	3 (0.0)	3 (0.0)	3 (0.0)
40	−	−	35 (0.0)	1 (0.0)*	2 (0.0)	3 (0.0)	3 (0.0)
42	+	−	30 (10.0)	1 (0.0)*	3 (0.0)	3 (0.0)	3 (0.0)
47	+	−	65 (7.7)	1 (0.0)*	2 (0.0)	3 (0.0)	3 (0.0)
**Total**	**25 (28.0)**	**25 (0.0)**	**827 (3.3)**	**14 (0.0)**	**67 (0.0)**	**52 (0.0)**	**69 (0.0)**

* Vaginal secretion samples from sheep with no history of recent abortion.

Table S2. Management practices of Q fever infected and non-infected control flocks in Eastern Province, Saudi Arabia.

Factors	No. of infected (cases = 25) flocks	No. of non-infected (control = 25) flocks
**Flock size**		
Small <290	9	16
Medium (290-500)	10	9
Large >500	6	0
**Purchase of breeding replacement**		
No	5	12
Yes	20	13
**Quarantine of purchased animals**		
No	20	17
Yes	5	8
**Animal exchange during breeding**		
No	4	18
Yes	21	7
**Contact with other sheep flocks**		
No	6	14
Yes	19	11
**Contact with other animals**		
No	2	7
Yes	23	18
**Lambing pen**		
No	16	9
Yes	9	16
**Recent history of abortion**		
No	0	25
Yes	25	0
**Change bedding after removing aborted materials**		
No	17	8
Yes	8	17
**Disinfect bedding after abortion**		
No	22	11
Yes	3	14

**Isolate aborted ewes**		
No	17	15
Yes	8	10
**Ticks on animals**		
No	11	19
Yes	14	6
**Ticks in environment**		
No	14	23
Yes	11	2
**Manure spreading**		
No	6	14
Yes	19	11
**History of Q fever**		
No	21	25
Yes	4	0
